# Translational Pharmacology

**DOI:** 10.3389/fphar.2017.00008

**Published:** 2017-01-19

**Authors:** Alastair G. Stewart

**Affiliations:** Department of Pharmacology and Therapeutics, University of MelbourneParkville, VIC, Australia

**Keywords:** mechanopharmacology, mechanobiology, pharmacodynamics (PD), pharmacokinetics (PK), PKPD modeling, bioassay profiling, translational pharmacology

The challenge for *Translational Pharmacology* is to improve the predictive value of the tools that are used to qualify the efficacy of a drug candidate. We should briefly consider the range of these tools and provide conjecture as to where the limitations may be and how these may be addressed. As this section of *Frontiers* develops, we will specifically welcome manuscripts that address ways of improving drug candidate qualification.

There is a crisis in science that extends to medical science and to translational pharmacology. The best publicized aspect of this complex crisis is in the widely acknowledged lack of reproducibility of research findings (Baker, 2016). Several authors have written about specific systematic attempts by Pharma (e.g., Amgen) to reproduce preclinical findings, with very low success rates being reported (Begley and Ellis, 2012). These failures are attributed to a variety of factors, including poorly specified methods, variable context-dependent behavior of tools and cell lines, inadequate experimental power, *post hoc* analyses, and other forms of significance finding (now commonly known as P hacking; Begley, 2013). The research community, including the editorial board of the Frontiers series, is actively addressing the concerns raised by these observations and the ensuing debate. More recently a damning view of clinical research as a whole, has highlighted that many studies are redundant or non-feasible, creating waste, and unnecessary risk to patients/volunteers (Ioannidis, 2016).

Closer to home we have some challenging failures in translational pharmacology. There have been several systematic analyses of the causes of major pharma pipeline attrition up to phase 2 of development. These studies consistently identify lack of efficacy as the cause of failure in approximately 50% of drug candidates (Cook et al., 2014). In this case, a “failure to fail” can be identified. Where can improvements in the process be found that will reduce the number of inefficacious candidates moving through to the later stages of clinical investigation? We have written an extensive commentary on one aspect of the drug candidate qualification process, namely the importance of cellular mechanics. We introduced the term “mechanopharmacology” to deal not only with the effects of drugs on mechanics, but also the effects of mechanics on drug actions (Krishnan et al., 2016), recognizing that the mechanical environment of the cells used in preclinical cellular pharmacology studies is often non-(patho)physiological. At a molecular level, the term also has utility in describing the impact of shear on molecular binding events and on protein shape.

The standard genomic-era processes often provide a target that has been identified through analyses of differentially expressed mRNA transcripts and somewhat less commonly via proteomics, particularly 2D differential in gel electrophoresis (2D-DIGE). The process of drug target validation would typically include the use of a gene-edited cell line or transient interference with siRNA to reduce protein expression, with phenotypic output of the assay system being assessed in the context of stimulating or inhibiting the function of the target protein. In the absence of any small molecule ligands, and presuming the target is intracellular and therefore not amenable to targeting by biologicals, a high throughput screen is the likely next step. However, if the target were in an accessible extracellular compartment then a biological approach may be as likely as a small molecule screening campaign. The assessment of efficacy is likely to be addressed in animal models and in a range of species, albeit that murine models are much more likely to be used than others. When considering the likely sources of discordance that lead to failures in efficacy in phase 2 clinical trials, the reliance on murine models looms large. There are a number of considerations. Is the pathology of the targeted condition faithfully reproduced? Has the model generated the “right outcome” but for different reasons than the human pathology it is intended to model. Is the model “too pure” in its construction. That is to say, are there natural exposures in the target human population that are not represented in the animal facility, which is environmentally controlled with SPF status that will influence the microbiome, an increasingly well-recognized influence on each of the major chronic diseases, including allergy (Marsland and Salami, 2015). Another feature of chronic diseases is the existence of co-morbidities that are related to the primary condition. However, often the animal model does not have a duration that allows these to be expressed. Indeed, many of the highest burden chronic diseases are associated with aging, or have more significant impact in aged patients, but it is usually prohibitively expensive to model the condition in aged mice.

Is there a solution for these widely acknowledged limitations in the processes that are used to qualify drug candidates? The recognition of these limitations is a useful starting point. Active consideration of the role that aging, infection/microbiome, co-morbid conditions, or the temporal development of chronic disease and genetic/epi-genetic background brings into focus possible strategies for risk mitigation. Thus, in my area of greatest interest in the lung, a disease such as COPD presents some significant challenges. The absence of any agents that affect the natural history of the disease (asides from smoking cessation adjunct therapies) attests to the difficulties. COPD is a disease associated with aging which typically develops after age 40, usually in individuals with a significant smoking history and then continues to progress after cessation of smoke exposure. Models in mice therefore are typically protracted (months) to achieve similar emphysematous changes to those in COPD patients, but I am not aware of any investigators that have been able to demonstrate a model that shows progressive lung function decline after smoking cessation. Evaluating drugs during the smoking phase therefore limits the breadth of potential applicability.

Thus, the grand challenge is to tackle the “failure to fail” by identifying strategies that have higher predictive value, but to do so in a cost and time effective manner. We think that some of the answers will come from the burgeoning area of cell/tissue/organ/disease-on-a-chip (Huh et al., 2013), as such models may be configured to use cells from affected patients of the right age and exposure history, obviating some of the more striking limitations of mouse models (Ingber, 2016). However, this technology is in its infancy, has limited benchmarking and has its own limitations in terms of a relative lack of cellular complexity and the usual criticisms of cell/organoid culture around dimensionality (2D vs. 3D), anatomical relationships and the deficiencies in the physiological integration due to the absence of neuroendocrine pathways. These challenges are being met by spectacular advances in disease-on-a-chip technology, promising improvements in lead qualification processes across the spectrum of safety, efficacy, and pharmacokinetic features that underwrite success in clinical progression of drug candidates.

The *Translational Pharmacology* section looks forward to receiving manuscripts that deal with drug target validation, structure-activity relationships, bioassay, and animal model development, with an emphasis on organ/disease-on-a-chip technology; biomarker discovery, definition and validation; pharmacodynamic/pharmacokinetic relationships; risk mitigation approaches in the discovery/preclinical interface, and other issues of relevance to the translational pathway.

## Author contributions

The author confirms being the sole contributor of this work and approved it for publication.

## Funding

The authors work cited in this commentary is supported by NHMRC project grant #1059665 and ARC LP160100635.

### Conflict of interest statement

The author declares that the research was conducted in the absence of any commercial or financial relationships that could be construed as a potential conflict of interest.
